# Digital detection of endonuclease mediated gene disruption in the HIV provirus

**DOI:** 10.1038/srep20064

**Published:** 2016-02-02

**Authors:** Ruth Hall Sedlak, Shu Liang, Nixon Niyonzima, Harshana S. De Silva Feelixge, Pavitra Roychoudhury, Alexander L. Greninger, Nicholas D. Weber, Sandrine Boissel, Andrew M. Scharenberg, Anqi Cheng, Amalia Magaret, Roger Bumgarner, Daniel Stone, Keith R. Jerome

**Affiliations:** 1 Department of Laboratory Medicine, University of Washington, Seattle, WA, 98195, USA; 2 Vaccine and Infectious Disease Division, Fred Hutchinson Cancer Research Center, Seattle, WA, 98102, USA; 3 Department of Biostatistics, University of Washington, Seattle, WA, 98195, USA; 4 Department of Microbiology, University of Washington, Seattle, WA, 98195, USA; 5 Seattle Children’s Research Institute, Seattle, WA, 98101, USA; 6 University of Washington School of Medicine, Seattle, WA, 98195, USA

## Abstract

Genome editing by designer nucleases is a rapidly evolving technology utilized in a highly diverse set of research fields. Among all fields, the T7 endonuclease mismatch cleavage assay, or Surveyor assay, is the most commonly used tool to assess genomic editing by designer nucleases. This assay, while relatively easy to perform, provides only a semi-quantitative measure of mutation efficiency that lacks sensitivity and accuracy. We demonstrate a simple droplet digital PCR assay that quickly quantitates a range of indel mutations with detection as low as 0.02% mutant in a wild type background and precision (≤6%CV) and accuracy superior to either mismatch cleavage assay or clonal sequencing when compared to next-generation sequencing. The precision and simplicity of this assay will facilitate comparison of gene editing approaches and their optimization, accelerating progress in this rapidly-moving field.

Genome editing has far-reaching applications across many fields, including treatment of human hereditary diseases^1^ and cure of viral diseases^2^,^3^,^4^. Many gene editing strategies rely on delivery of sequence-specific designer nucleases (zinc finger nucleases, meganucleases, TALENs, megaTALs, and CRISPR-Cas enzymes)^5^,^6^,^7^. These nucleases induce various deletion, insertion, or SNP mutations within a target sequence. Characterizing and enumerating these mutations is an integral step in the genome editing research process that drives these nuclease technologies forward.

The genome editing field widely utilizes the enzyme mismatch cleavage assay, commonly called Surveyor or T7 endonuclease I mismatch cleavage assay (T7 MCA), to experimentally quantify mutation rates in samples treated with designer nucleases^8^,^9^. This method detects unknown mutations by identifying heteroduplex DNA formed subsequent to melting and hybridizing mutant and wild type alleles. While this method is highly effective for screening large numbers of samples relatively quickly and easily, it is only semi-quantitative and prone to subjective analysis. Other methods rely on sequencing, either traditional Sanger sequencing of clonal amplicons or next-generation sequencing (NGS), of nuclease target regions. Sequencing methods are effective and quantitative, but are more costly in terms of time, reagents, and specialized equipment.

A heretofore under-explored method for accurate and fast quantitative mutation detection employs droplet digital PCR (ddPCR). ddPCR divides a single PCR reaction into thousands of nanoliter droplets containing TaqMan hydrolysis probes and target DNA sequences^10^,^11^. These reactions are thermocycled to endpoint to determine the absolute quantity of target DNA. Here we compared the standard genome editing laboratory mutation detection methods (T7 MCA, clonal amplicon sequencing, and NGS) with a novel method of mutation quantitation by droplet digital PCR.

## Material and Methods

### Cell culture

SupT1 cells (ATCC# CRL-1942) were grown in RPMI 1640 (Life Technologies) supplemented with 10% FBS. HEK293^12^ and 293T (ATCC# CRL-3216) cells were grown in DMEM (Life Technologies) supplemented with 10% FBS.

### HIV *pol*-specific megaTALs

**Figure 1:**
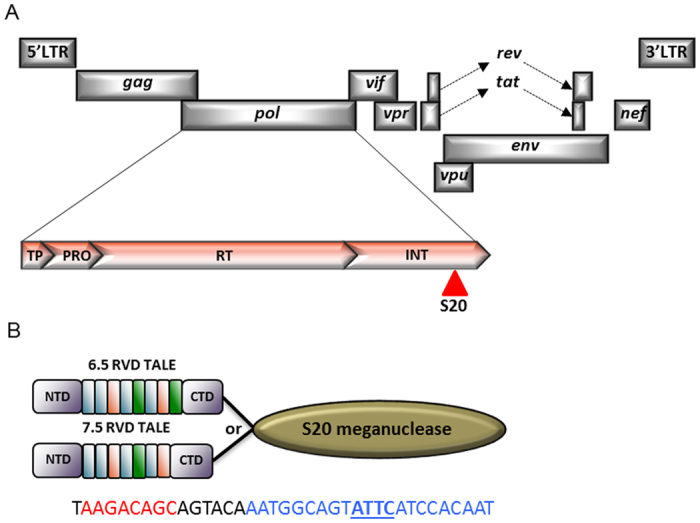
Cleavage of HIV *pol* by an engineered megaTAL. (**A**) Location of the megaTAL cleavage site (S20, red triangle) within the HIV provirus. (**B**) Tal effector (TALE) and meganuclesase domains for the HIV *pol*-specific 6.5 or 7.5 RVD repeat containing megaTALs alongside their HIV *pol* target sequences. TALE binding (red), S20 meganuclease binding (blue) and S20 meganuclease cleavage (underlined) sites are shown. NTD – N-terminal domain; CTD – C-terminal domain.

Two megaTAL endonucleases specific for an HIV *pol* sequence encoding the HIV integrase (Fig. 1) were generated using a previously described approach^13^,^14^. Briefly, a I-*OnuI* based meganuclease (S20) specific for a 22bp target sequence in the HIV *pol* gene that was selected by yeast surface display^15^ was obtained from Bluebird Bio. TAL effector arrays containing 6.5 or 7.5 repeat variable diresidue (RVD) repeats that recognize 6 or 7 nucleotides respectively 5′ to the S20 target site were fused to the N-terminus of S20 using a 4 amino acid VGGS Zn4 linker sequence to generate each megaTAL.

### Plasmids

Plasmids pDHIV3 and pDHIV3-GFP have been described previously^16^,^17^. Briefly, pDHIV3 contains an env-deleted, replication-incompetent HIV genome derived from NL4-3, and pDHIV3-GFP also contains a green fluorescent protein (GFP) reporter gene in place of *nef*. The plasmid pscAAV-CMV-Trex2 expressing the three prime to 5 prime exonuclease 2 (Trex2) from the CMV promoter has been described previously^2^. The megaTAL expression plasmids pRRL-6.5-HIV*pol*-megaTAL and pRRL-7.5-HIV*pol*-megaTAL were generated by linking the gene for each megaTAL to the BFP reporter gene using a T2A sequence in a lentivirus vector plasmid so that gene expression was driven by the SFFV LTR promoter.

### Mutant ddPCR control plasmids

**Figure 2:**
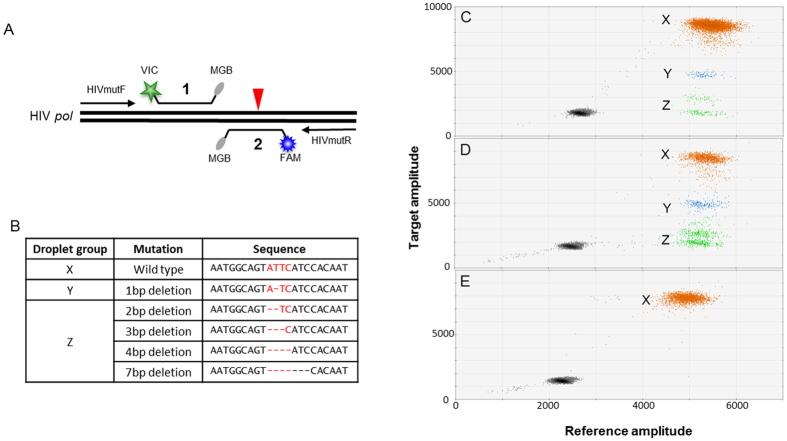
Droplet digital PCR can detect a wide range of deletion mutations. (**A**) Primer and probe design. HIV *pol*-specific forward and reverse primers are used with a reference (1) and target (2) probe that bind to opposite strands of the same PCR amplicon. The target probe (2) is centered on the megaTAL target site, indicated by a red triangle. (**B**) Target site deletion mutations used for ddPCR assay validation. (**C**) Two dimensional ddPCR amplitude plot showing that the assay detects the reference sequence in addition to wild type target sequence (X), one base pair deletions (Y), or 2, 3, 4 and 7 base pair deletions (Z) at the megaTAL target site in reference plasmids. Mutant control plasmids were spiked into a background of wild type plasmid at an approximate ratio of 85:1:2 (X:Y:Z). Droplets containing no target are shown in gray. (**D**) Two dimensional ddPCR amplitude plot showing that the assay detects the reference sequence, wild type (X), and mutant (Y, Z) sequences in 293T cells 72 hours after transfection with pDHIV3 and plasmids expressing the 6.5 megaTAL and Trex2. (**E**) 293T cells with pDHIV3 and no megaTAL treatment (negative control) have a very low false mutation rate; >99% of reference positive droplets are also target positive (X).

Initial ddPCR assay development was performed on plasmids containing 1, 3 and 7 base pair deletion mutations that were identified at the megaTAL target site in a pilot experiment using SupT1 cells containing an integrated copy of DHIV3-GFP that were transduced with the RRL-7.5-HIV*pol*-megaTAL lentiviral vector (Supplemental Fig. 1). Additional plasmids were generated that contain the wild type target sequence and 2 or 4 base pair deletions (Fig. 2B). Plasmids were created by Gibson assembly^18^ using the Gibson Assembly Cloning Kit (New England Biolabs). Briefly, a 3 piece Gibson Assembly was performed using KpnI linearized pGEM-7ZF (Promega) and 2 PCR products consisting of the left or right end of the ddPCR HIV *pol* PCR amplicon. Complimentary left and right end PCR products containing wild type or mutant target sequences introduced into the overlapping regions were amplified from the plasmid pDHIV3 using the primers described in Supplemental Table 1. Wild type and mutant amplicon sequences were confirmed by Sanger sequencing using M13F or M13R primers.

### T7 endonuclease I mismatch cleavage assay

PCR amplicons spanning the HIV-*pol* megaTAL target site were generated using Phusion polymerase (New England Biolabs), and primers HIVintF (TAGCAGGAAGATGGCCAGTA) and HIVintR (TCCTGTATGCAGACCCCAAT). PCR products were column purified before 200–400ng of DNA was heteroduplexed by heating to 98^o^C for 10 minutes and then slowly cooling to room temperature before placing on ice. Digestions were performed in a volume of 15 μl with 5U of T7 endonuclease I at 37^o^C for 30 minutes before cleavage was analysed on a 2% agarose gel. Quantification of cleavage was perfomed using ImageJ software as previously described^3^.

### HIV *pol* integrated target site controls

In order to generate control cells with integrated copies of wild type or mutant target sites a series of lentiviral vectors were generated. Fragments spanning the ddPCR HIV *pol* target sequence were cloned into a lentiviral GFP reporter vector by Gibson Assembly. Briefly, PCR amplicons containing wild type, 1 or 7 base pair deletions were amplified from the HIV-*pol* ddPCR control plasmids using primers Lenti-polF (GCTTGATATCGAATTCCCACCTTGGTAGCAGTTCATGTAG) and Lenti-polR (CTCTGTTCCTACGCGTCCAAAATCCTCATCCTGTCTACTT). For each PCR fragment a Gibson Assembly was then performed with BstXI-linearized pRRL-MND-GFP so that the ddPCR target sequence was introduced between the HIV cPPT sequence and the MND promoter driving expression of GFP. To generate a VSV-G pseudotyped stock of each lentiviral vector a 10cm dish containing 293T cells at 70% confluence was transfected with a total of 10 μg of lentivirus vector plasmid and the packaging plasmids psPAX2 and pMD2G. At 72 hours post transfection cell supernatants were collected and stored at −80^o^C. Control SupT1 cells with integrated copies of wild type or mutant ddPCR target sites were then generated by transduction with each lentivirus vector. Genomic DNA was extracted from bulk infections so that ddPCR would be performed on control samples with a polyclonal integration site profile. To ensure that the provirus copy number remained below 1 copy/cell lentivirus transduction levels of less than 30% were chosen^19^. SupT1 cells were infected with equalized GFP-expressing units of each vector, expanded for 5 days, then sorted for GFP-positive cells before genomic DNA extraction using the Qiaqen DNeasy blood and tissue DNA kit.

### Clonal PCR amplicon sequencing

Clonal amplicon sequencing was performed on DNA extracted from treated cells as previously described^3^. Briefly, megaTAL target sites were amplified using Phusion polymerase (New England Biolabs), and primers HIVintF (TAGCAGGAAGATGGCCAGTA) and HIVintR (TCCTGTATGCAGACCCCAAT). PCR products were sub-cloned using the Zero Blunt TOPO PCR cloning kit (Life Technologies). TOPO-cloned PCR products were transformed into One Shot Top10 *Escherichia coli* (Life Technologies) for clonal analysis and individual colonies were picked for plasmid purification from which the clonal megaTAL target sites were sequenced using T7 or SP6 sequencing primers.

### Analysis of mutation rates for plasmid-derived HIV in megaTAL treated cells

293T cells were plated in 12 well plates at 2 × 10^5^ cells/well and the following day transfected with pDHIV3, in the presence or absence of the megaTAL and Trex expressing plasmids pRRL-6.5-HIV-pol, pRRL-7.5-HIV-pol and pscAAV-CMV-Trex2 as indicated (Fig. 2). Each well was transfected with 1 μg of each plasmid and a total of 3 μg DNA. At 72 hours post transfection cells were harvested and genomic DNA was extracted using the DNeasy blood and tissue DNA kit (QIagen). Mutations were quantified by T7 endonuclease I cleavage assay, clonal PCR amplicon sequencing, Illumina sequencing or ddPCR.

### Analysis of integrated HIV provirus mutation rates in megaTAL-treated cells

HIV provirus mutation rates were analysed in SupT1 cells infected with the GFP-expressing, VSV-G pseudotyped DHIV3-GFP virus that is replication incompetent. SupT1 cells were infected with DHIV3-GFP at <30% (as determined by flow cytometry for GFP expression) to ensure that the provirus copy number remained below 1 copy/cell. GFP positive SupT1 cells were sorted and then transduced with VSV-G pseudotyped 6.5-HIV*pol*-megaTAL and 7.5-HIV*pol*-megaTAL lentiviral vectors at equalised levels so that ~80% of cells were GFP+/BFP+ at 24 hours post transduction. Cells were harvested at 72 hours post transfection and genomic DNA was extracted using the DNeasy blood and tissue DNA kit (QIagen). Mutations were quantified by T7 endonuclease I cleavage assay, clonal PCR amplicon sequencing, Illumina sequencing or ddPCR.

### Droplet digital PCR (ddPCR) assay design and reaction conditions

A single primer set and two Taqman hydrolysis probes, one for target (wild type) sequence and one for HIV reference sequence, were designed using Primer Express 3.0 (Life Technologies). The target probe is complementary to the region containing the endonuclease target site, while the reference probe is complementary to a portion of the HIV genome outside the endonuclease target site (Fig. 2A). When a deletion or missense mutation occurs, the target probe cannot bind the mutant sequence, but the reference probe binding is not disrupted. Mutant sequences are observed as reference-positive, target-negative droplets (Fig. 2C,D)Y and Z droplet populations). The mutation rate of a sample is determined by the following equation: (target negative droplets/total reference positive droplets) *100. Droplet digital PCR reactions were run on the QX100 Droplet Digital PCR System (Bio-Rad Laboratories, Hercules, CA). The primer and probe sequences are as follows: HIVmutF 5′- GGACAGGTAAGAGATCAGGCTGA-3′, HIVmutR 5′- CCAATCCCCCCTTTTCTTTTA-3′, reference probe 5′- VIC-CATCTTAAGACAGCAGTACAA-MGBNFQ-3′, target probe 5′-6FAM-TTGTGGATGAATACTGC-MGBNFQ-3′. The ddPCR Supermix for Probes (no dUTP) (Bio-Rad Laboratories) was mixed with 900nM primers, 250nM probes, and varying volumes of template DNA and water to achieve 20 μl total reaction volumes. Reactions were packaged into droplets according to manufacturer’s instructions and thermocycled with the following conditions: 95 °C for 10 minutes, 40 cycles of 94 °C for 30 seconds and 57 °C for 1 min, followed by 10 minutes at 98 °C and a 10 °C hold. These conditions were established with a gradient PCR (50 °C to 60 °C) on plasmid DNA with known mutations to determine the optimal amplitude difference between target positive and target negative droplets. HindIII (New England Biolabs) was used to digest gDNA samples according to manufacturer’s recommendation, 4-6 hours at 37 °C in either 20 μl or 50 μl volumes with 10X Cutsmart buffer (New England Biolabs).

### Determination of ddPCR Limit of Blank (LoB), Limit of Detection (LoD), and Limit of Quantification (LoQ)

LoB was estimated with 60 measurements under the guidelines of established protocol^20^ (E-17A2 NCCLS). Control SupT1 cells with integrated copies of wild type ddPCR target sites were diluted with 10 mM Tris pH 8 into a 5 level dilution series, and 4 replicates were run per day on 3 separate days. The LoB was determined non-parametrically as the 95^th^ percentile of mutation rates from samples with no added mutant DNA.

To establish LoD and LoQ, control SupT1 cells having integrated copies of mutant ddPCR target sites were mixed with control SupT1 cells having integrated copies of wild type ddPCR target sites, and diluted with 10 mM Tris pH 8. The resulting targeted mutation rates ranged between LoB to five times LoB; and these were replicated six times on each of 2 days. LoD was determined as the point ~2 standard deviations above LoB; and LoQ was the mutation rate at which the coefficient of variation fell below 20%.

### lllumina sequencing and analysis

For library preparation of research samples, a pair of amplicon primers with adapter sequences of 16S ribosomal RNA gene (16S rRNA) were designed (see Supplemental Table 3) to amplify a 327bp HIV sequence surrounding the TaqMan amplicon site using Kappa HiFi polymerase or Phusion HiFi DNA polymerase (KAPAbiosystems, NEW ENGLAND *BioLabs*). Purified PCR products were diluted to 1ng/uL and quarter-volume NexteraXT reactions performed, following manufacturer’s protocol (Illumina) with a sample volume of 1.25 uL. Libraries were amplified and barcoded using 14 cycles of PCR with the NexteraXT Index Kit (Illumina). Samples were pooled and quantitated by Bioanalyzer (Agilent) such that approximately 200,000 reads were achieved per sample on a MiSeq sequencer (Illumina).

Raw reads were pre-processed using tools from the Galaxy suite^21^. Reads were trimmed using Trimmomatic^22^ and Cutadapt^23^ to remove adapter contaminants and low-quality regions (Q < 30) at the 3′ and 5′ ends; any remaining reads shorter than 100 bp were discarded. Trimmed reads were mapped to the DHIV reference sequence using Bowtie2^24^ and exported for further analysis. Variant analysis was performed using a custom script that used functions from the Rsamtools, ShortRead and Biostrings packages in R/Bioconductor^25^,^26^,^27^. Aligned reads were scanned for insertions, deletions and SNPs, focusing in particular on regions identified as the endonuclease target region (‘tgt’, 17 bp), forward and reverse primer binding regions (‘fwd’, 23 bp and ‘rev’, 21 bp respectively) and reference region (‘ref’ 21 bp). Any reads that did not cover all four regions were discarded. Quality scores of insertions were also recorded to identify poor quality calls and the percentages of reads containing mutations of each type and in each region were counted.

## Results

### Detection of megaTAL-mediated HIV *pol* mutations by droplet digital PCR (ddPCR)

**Figure 3:**
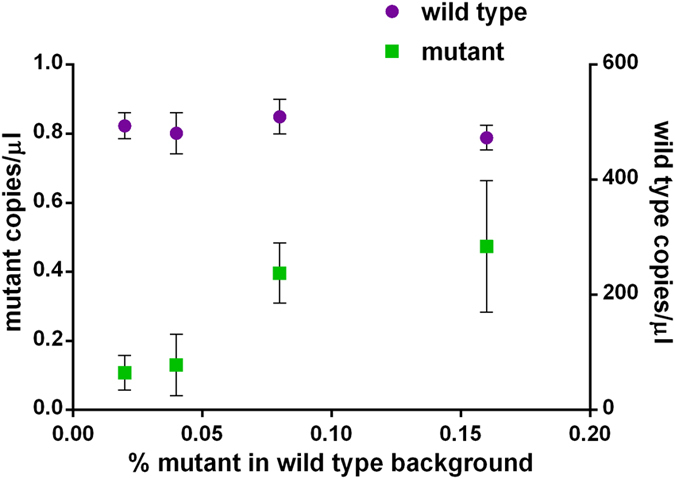
Lower range of detection for a mutant plasmid (2 base pair deletion) in a background of wild type plasmid.Each point represents the mean of 3 reactions. All reactions were non-negative for mutant down to 0.02% mutant. Error bars indicate one standard deviation.

To assess the potential benefits of droplet digital PCR (ddPCR) in accurately and reproducibly quantifying mutation rates in megaTAL treated cells, a ddPCR assay targeting the megaTAL cleavage site (Fig. 1) was designed. This assay utilized a single primer set and two TaqMan hydrolysis probes on opposite DNA strands, one annealing over the megaTAL target site (target probe) and the other annealing at an upstream portion of the HIV *pol* gene expected to be unaffected by megaTAL induced cleavage and mutation (reference probe) (Fig. 2A). This ddPCR assay was optimized on plasmid controls designed with 1, 2, 3 4 or 7 base pair mutations at the megaTAL target site within HIV *pol* (Fig. 2B,C). Droplets positive for target and reference sequence (Fig. 2C, X droplet population) contain unmutated, wild type HIV proviral sequence. Droplets negative for target sequence but positive for reference sequence (Fig. 2C, Z droplet population) represent HIV proviral sequences with 2, 3, 4 or 7 base pair mutations. The droplet population with a target amplitude between the wild type and mutant droplets (Fig. 2C, Y droplet population) represent sequences containing a 1 base pair deletion at the megaTAL target site which significantly reduces the efficiency of target probe binding. The lower detection limit on plasmid samples was assessed by spiking a 2-fold dilution series of plasmid containing the 2 base pair deletion into a constant background of wild type plasmid. Detection as low as 0.02% mutant plasmid in a background of wild type plasmid was achieved (Fig. 3).

### Limit of Blank, Limit of Detection, and Limit of Quantification of ddPCR on genomic samples

**Figure 4:**
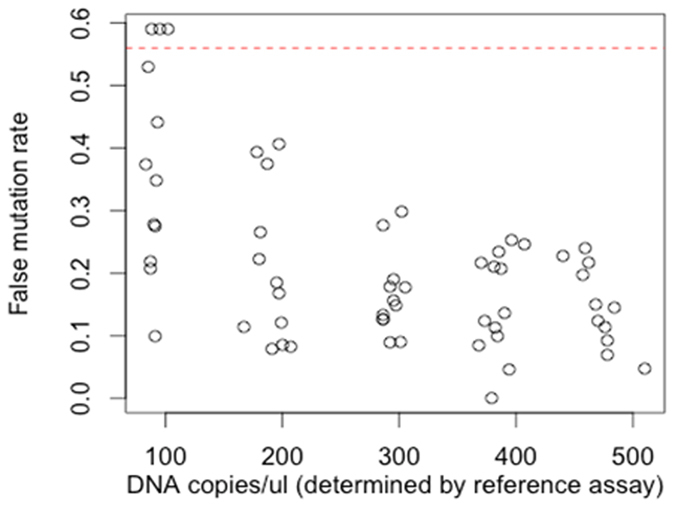
Scatter plot of false mutation rate detected from reference cell line genomic DNA (SupT1-HIV-WT sample). The red dashed line indicates the empirically determined Limit of Blank (LoB).

To simulate the reaction milieu when testing megaTAL-treated research samples, the detection limit was subsequently assessed using genomic DNA samples containing integrated HIV with mutant and wild type sequences at the megaTAL target site. The Limit of Blank (LoB) (false mutation rate or background) observed in a sample of wild type HIV integrated genomic DNA was 0.56% (Fig. 4).

Though the assay could detect mutations well below the 0.56% LoB, as observed with plasmid DNA (Fig. 3), the false mutation rate limits the effective LoD on cellular samples to 1.06% mutant in a wild type background. The LoQ for this assay is 2.19% with a 20% CV.

### Comparison of mutation detection methods in cells containing HIV provirus treated with megaTALs

#### Droplet digital PCR

**Figure 5:**
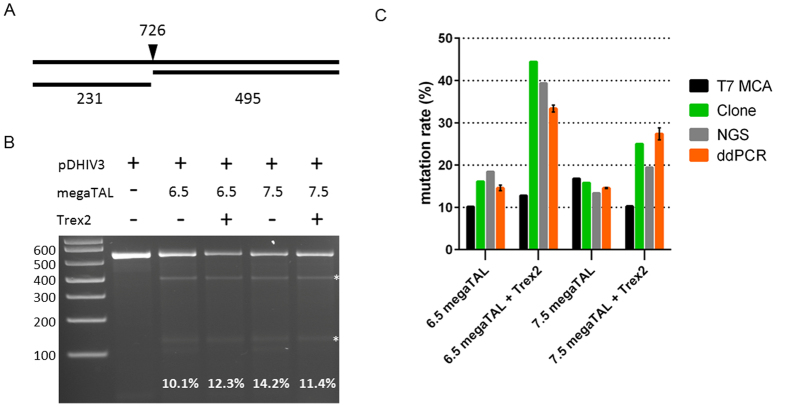
Mutation detection assay comparison **(A)** T7 endonuclease I MCA mutation detection. S20 target site-specific PCR amplicon size and predicted cleavage products.(**B**) 293T cells were transfected with pDHIV3 and plasmids expressing the indicated megaTALs +/− a Trex2 expressing plasmid. At 72 hours post transfection DNA was extracted and PCR amplicons spanning the target site were amplified for analysis of target site-disruption. (**C**) Comparison of the T7 mismatch cleavage assay (T7 MCA), clone sequencing (clone), illumina sequencing (NGS), and droplet digital PCR (ddPCR) mutation detection in megaTAL treated 293T cells transfected with pDHIV3 +/− Trex2 treatment. Error bars for ddPCR represent the standard deviation of three replicate samples.

After validation of the ddPCR assay on plasmids and cell lines with known mutations, cells transfected with plasmids containing HIV sequence and plasmids expressing megaTALs with or without a Trex2 expressing plasmid were cultured to induce genome disrupting mutations in the HIV provirus. The HIV *pol* mutation rates in these cell lines were interrogated by ddPCR (Figs 2D, 5C). The droplet populations observed mirrored the droplet populations evident in control DNA with known deletions of 1, 2, 3, 4 and 7 base pairs (Fig. 2C). Mutation rates were calculated based on the number of target negative, reference positive droplets (mutant HIV) divided by the total reference positive droplets (total HIV). Mutation rates ranged from 14.07% to 33.05%, with the megaTAL + Trex2 samples exhibiting higher mutation rates than the megaTAL only samples (Fig. 5C). Intra- and interassay variability were low, ranging from 3.2–5.9% CV and 1.6–8.8% CV, respectively (Table 1).

#### T7 endonuclease I mismatch cleavage assay

The HIV mutation rates at the megaTAL target site were assessed with the T7 endonuclease 1 mismatch cleavage assay (T7 MCA) (Fig. 5). All cultures treated with megaTALs, with or without Trex2, exhibited mutation rates detectable by T7 MCA (10–14%). No cleavage band was observed in gDNA from untreated control cells. The T7 MCA was repeated twice with varying levels of template DNA and yielded similar mutation rates between the duplicates. The mutation rates reported by T7 MCA were much lower than those detected by ddPCR in several samples (Fig. 5C).

#### Clonal PCR amplicon sequencing

The megaTAL target site in the treated cells was amplified and cloned into cells for clonal Sanger sequencing. Between 19 and 40 clones were sequenced for each megaTAL treatment. The number of mutant clones/the number of wild type clones determined the mutation frequency, which ranged from 15.79% to 44.44% (Fig. 5C). These values correlated well with the ddPCR mutation rates.

#### Next-generation sequencing (NGS)

The *pol* mutation rates were additionally interrogated in the megaTAL-treated cell lines by Illumina NGS. Mutation rates determined by NGS ranged from 17.9% to 45.9% and correlated well with rates measured by ddPCR (Fig. 5C). The NGS data was further analysed to determine the rates of mutations present that could affect the primer or reference probe binding regions of the ddPCR assay (Fig. 2A), because mutations in these areas would hinder detection of mutations at the target site due to the absence of amplification or reference probe binding. The mutation rates in these regions, called binding region indels (BR-indels), ranged from 2.1% to 4.1% (Table 1), indicating that mutations in these regions could account for potential mutation rate underestimation by ddPCR.

### Analysis of integrated HIV provirus mutation rates in megaTAL-treated cells

In the previous mutation analysis comparison there were relatively high levels of HIV target sequences from which to discern a megaTAL-induced mutation rate. To test how these methods compare on a sample that more closely mimics HIV biology, the mutation analysis methods were compared on a cell line containing integrated HIV (~5% cells containing integrated HIV, determined by ddPCR). SupT1 cells containing integrated DHIV3 were treated with the megaTALs and assayed by all four mutation detection methods (Table 2). In the treated samples, the T7 endonuclease mismatch cleavage assay was unable to detect mutations in any of the conditions tested. By the other methods, cells treated with megaTAL 7.5 exhibited a consistently higher mutation rate (6.5%, 5.75% and 8.59% by clonal sequencing, NGS, and ddPCR respectively) than cells treated with megaTAL 6.5 (1.3%, 2.08% and 3.98%). Control cells without megaTAL treatment showed background levels of mutation by all assays.

## Discussion

We have designed a droplet digital PCR assay that delivers reproducible, low-level detection of many varied deletion and insertion mutations within an endonuclease-treated HIV proviral sequence. Mutation detection as low as 0.02% was observed, with a statistically determined limit of detection of 1.06%. In samples containing relatively fewer targets for ddPCR (such as the experiment in Table 2, where only about 5% of cells contained the target integrated HIV, or the results with low levels of input DNA in Fig. 4), ddPCR background levels can be slightly higher. While these background levels are somewhat higher than published allele-specific SNP assays, such as for the well characterized BRAF^v600E^ mutation^28^,^29^, our ddPCR approach has the distinct advantage of quantifying a wide range of insertions and deletions rapidly.

The T7 MCA, the workhorse assay for labs performing gene-editing experiments, is also a rapid method that detects a wide range of insertion and deletion mutations^8^,^9^. However, drawbacks of this method include poor accuracy and precision, with only semi-quantitative results and a certain level of subjectivity in the data analysis. When analyzing cleavage bands on a gel with software, bias can come from two sources. The uncleaved bands on the gel can have high density that overwhelms the peaks of other bands, leading to underestimation of the fraction cleaved. Also, since the threshold setting is normally performed manually, the result of the density of each peak is arbitrary. Indeed, our data show that droplet digital PCR is a much more accurate mutation screening tool than the T7 MCA (Fig. 5C).

Moreover, the ddPCR assay agrees well with traditional and next-generation sequencing (NGS) methods, but has the advantage of being performed more easily and rapidly than any sequencing method. Clonal sequencing requires multiple cloning and purification steps before Sanger sequencing, which leads to longer turnaround time. Also, mutation rate data is only as accurate as the number of clone sequencing replicates performed. It is often not feasible to sequence more than 50–100 clones, which severely limits the analysis. NGS offers highly sensitive detection with very low background while providing specific information about the mutations detected and is now considered the gold standard in this field. However, Illumina sequencing is not suitable for routine performance in research labs as it is still expensive to perform routinely and requires multiple library preparation steps, which leads to a long turnaround time. In addition, sophisticated computation analysis tools are required to interpret the large volumes of data provided by NGS. A recently-described method used in some gene-editing laboratories is TIDE, Tracking of Indels by Decomposition^30^. While not directly compared here, this method tends to underreport nonhomologous end joining events, so would be at a disadvantage compared to ddPCR for quantitative applications.

The ddPCR method described here is highly accurate and precise, but at the same time easily performed, and can easily be adapted to additional target sites of interest. The precision and simplicity of this assay will allow comparison of gene editing approaches and their optimization, facilitating accelerating progress in this rapidly-moving field.

## Additional Information

**How to cite this article**: Sedlak, R. H. *et al*. Digital detection of endonuclease mediated gene disruption in the HIV provirus. *Sci. Rep.* **6**, 20064; doi: 10.1038/srep20064 (2016).

## Supplementary Material

Supplementary Information

## Figures and Tables

**Table 1:** Coefficient of variation (CV) and mutation rate within the primer binding regions for the ddPCR assay on megaTAL treated samples.

**293T pDHIV3 transfected cell treatments**	**ddPCR CV (%)**		**BR-Indels**
	**A**	**B**	
6.5 megaTAL	5.2	6.5	2.3
6.5 megaTAL + Trex2	3.2	2.3	2.8
7.5 megaTAL	3.5	8.8	1.5
7.5 megaTAL + Trex2	5.9	1.6	2.0

A is intraassay and B is interassay variability based on two separate runs of 3 reactions per run for each sample. BR-Indels (binding region insertion-deletions) are mutations that affect either of the two primer binding regions or reference probe binding region in sequences that have a deletion or insertion in the target binding site, representing target site mutations identified by NGS that would not be detected by ddPCR.

**Table 2:** Comparison of mutation detection methods in SupT1 cells containing integrated HIV provirus treated with 6.5 megaTAL or 7.5 megaTAL.

**SupT1 HIV integrated cell line treated with meganuclease**	**T7 MCA**	**Clone**	**NGS**	**ddPCR**	**ddPCR CV (%)**	**BR-Indels**
untreated	NC	0	0.01	1.62	15.8	0.00
6.5 megaTAL	NC	1.3	2.08	3.98	21.4	0.04
7.5 megaTAL	NC	6.5	5.75	8.59	13.8	0.20

NC is no cleavage band visible. T7 mismatch cleavage assay (T7 MCA), clone sequencing (clone), illumina sequencing (NGS) and ddPCR. BR-Indels (binding region insertion-deletions) are mutations that affect either of the two primer binding regions or reference probe binding region in sequences that have a deletion or insertion in the target binding site, representing target site mutations identified by NGS that would not be detected by ddPCR.
