# Supplementary material for: Digital detection of endonuclease mediated gene disruption in the HIV provirus
Source: Sci Rep. 2016 Feb 2;6:20064. doi: 10.1038/srep20064 (PMC4735761; doi:10.1038/srep20064)

Supplemental Information

Digital detection of endonuclease mediated gene disruption in the HIV provirus

Ruth Hall Sedlak[#1], Shu Liang[#1], Nixon Niyonzima[2], Harshana S. De Silva Feelixge[2], Pavitra Roychoudhury[2], Alexander L. Greninger[1], Nicholas D. Weber[2‡], Sandrine Boissel[5], Andrew M. Scharenberg, Anqi Cheng[3], Amalia Magaret[2, 3], Roger Bumgarner[4], Daniel Stone[2], Keith R. Jerome*[1,2]

Supplemental Table 1. Primers for Gibson assembly fragment preparation. The megaTAL target site and mutations are highlighted in red. The sequences underlined are the overlapping part of the two fragments. The sequences in blue are the complementary sequences for pGEM-7ZF

Primer Name	Sequence
Primer WT	AATGGCAGTATTCATCCACAATTTTAAAAGAAAAGGGGGGATTGGG
Primer WT'	TAAAATTGTGGATGAATACTGCCATTTGTACTGCTGTCTTAAGATG
Primer 1	AATGGCAGTAT-CATCCACAATTTTAAAAGAAAAGGGGGGATTGGG
Primer 1'	TAAAATTGTGGATG-ATACTGCCATTTGTACTGCTGTCTTAAGATG
Primer 2	AATGGCAGT--TCATCCACAATTTTAAAAGAAAAGGGGGGATTGGG
Primer 2'	TAAAATTGTGGATGA--ACTGCCATTTGTACTGCTGTCTTAAGATG
Primer 3	AATGGCAG---TCATCCACAATTTTAAAAGAAAAGGGGGGATTGGG
Primer 3'	TAAAATTGTGGATGA---CTGCCATTTGTACTGCTGTCTTAAGATG
Primer 4	AATGGCAGT----ATCCACAATTTTAAAAGAAAAGGGGGGATTGGG

Primer 4’	TAAAATTGTGGAT----ACTGCCATTTGTACTGCTGTCTTAAGATG
Primer 7	AATGGCAGT-------CACAATTTTAAAAGAAAAGGGGGGATTGGG
Primer 7’	TAAAATTGTG--------ACTGCCATTTGTACTGCTGTCTTAAGATG
Primer A	CTCTAGACTCGAGGAATTCG*CTTGGTAGCAGTTCATGTAG*
Primer B	TTATCGATTTCGAACCCGGG*AATCCTCATCCTGTCTACTT*

Supplemental Table 2 Amplicon primers for Illumina sequencing library preparation. The 16S adapter sequences are underlined.

Primer	Sequence
Illseq_Fwd	<u>TCGTCGGCAGCGTCAGATGTGTATAAGAGACAG</u>ACAGTTAAGGCCGCCTGTTG
Illseq_Rev	<u>GTCTCGTGGGCTCGGAGATGTGTATAAGAGACAG</u>TGGATCTCTGCTGTCCCTGT

Supplemental Figure 1. Mutation detection in 7.5-HIV*pol*-megaTAL treated DHIV3-GFP infected SupT1 cells. SupT1 cells containing 0.056 integrated copies of DHIV3-GFP per cell were transduced with a lentivirus vector expressing BFP and the 7.5-HIV*pol*-megaTAL. Levels of lentivirus transduction (A), mutation frequency by T7 endonuclease I MCA assay (B) and mutation frequency by amplicon sequencing (C) were monitored at 72 hours post transduction. Red = SNP; - = deletion.

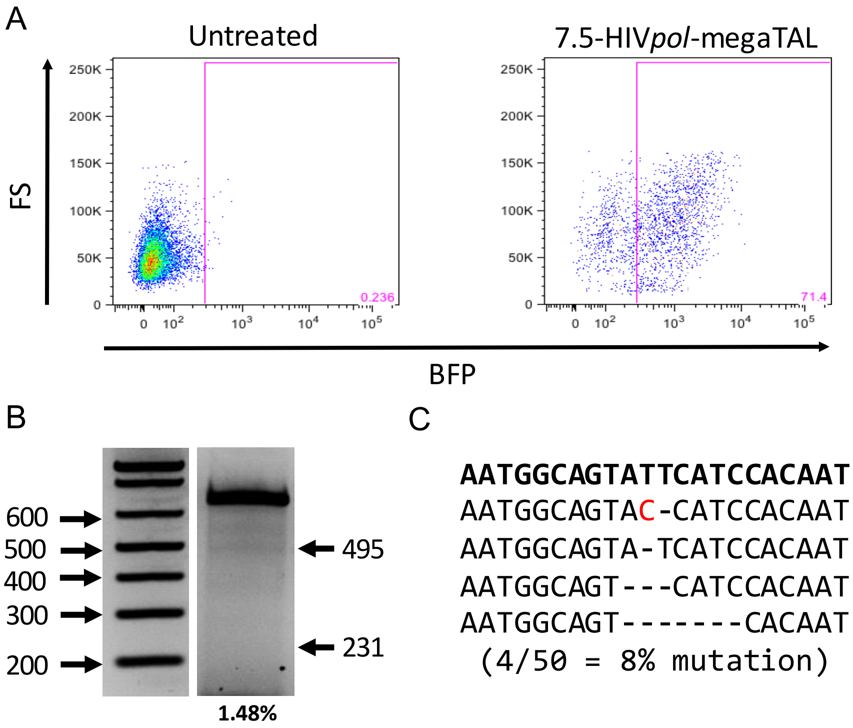

Supplement: Supplementary Information [file srep20064-s1.pdf]
